# Treatment of Synthetic Wastewater Containing Polystyrene (PS) Nanoplastics by Membrane Bioreactor (MBR): Study of the Effects on Microbial Community and Membrane Fouling

**DOI:** 10.3390/membranes14080174

**Published:** 2024-08-09

**Authors:** Anamary Pompa-Pernía, Serena Molina, Laura Cherta, Lorena Martínez-García, Junkal Landaburu-Aguirre

**Affiliations:** 1 IMDEA Water Institute, Avenida Punto Com, 2, Alcalá de Henares, 28805 Madrid, Spain; serena.molina@imdea.org (S.M.); laura.cherta@imdea.org (L.C.); lorena.martinez@imdea.org (L.M.-G.); junkal.landaburu@imdea.org (J.L.-A.); 2 Chemical Engineering Department, University of Alcalá, Ctra. Madrid-Barcelona Km 33.600, Alcalá de Henares, 28871 Madrid, Spain

**Keywords:** activated sludge, membrane bioreactor, membrane fouling, microfiltration, microbial community, nanoplastics, Py-GC-MS, ultrafiltration recycled membrane

## Abstract

The persistent presence of micro- and nanoplastics (MNPs) in aquatic environments, particularly via effluents from wastewater treatment plants (WWTPs), poses significant ecological risks. This study investigated the removal efficiency of polystyrene nanoplastics (PS-NPs) using a lab-scale aerobic membrane bioreactor (aMBR) equipped with different membrane types: microfiltration (MF), commercial ultrafiltration (c-UF), and recycled ultrafiltration (r-UF) membranes. Performance was assessed using synthetic urban wastewater spiked with PS-NPs, focusing on membrane efficiency, fouling behavior, and microbial community shifts. All aMBR systems achieved high organic matter removal, exceeding a 97% COD reduction in both the control and PS-exposed reactors. While low concentrations of PS-NPs did not significantly impact the sludge settleability or soluble microbial products initially, a higher accumulation increased the carbohydrate concentrations, indicating a protective bacterial response. The microbial community composition also adapted over time under polystyrene stress. All membrane types exhibited substantial NP removal; however, the presence of nano-sized PS particles negatively affected the membrane performance, enhancing the fouling phenomena and increasing transmembrane pressure. Despite this, the r-UF membrane demonstrated comparable efficiency to c-UF, suggesting its potential for sustainable applications. Advanced characterization techniques including pyrolysis gas chromatography/mass spectrometry (Py-GC/MS) were employed for NP detection and quantification.

## 1. Introduction

The widespread use of plastics has led to a global pollution crisis, with detrimental impacts on ecosystems. Of particular concern are micronanoplastics (MNPs), raising questions about their effects on aquatic environments and the need for effective mitigation strategies [1]. MNPs are classified as primary (intentionally manufactured) or secondary (formed from the breakdown of larger plastics). Regarding their sizes, MPs are fragments smaller than 5 mm, and nanoplastics (NPs) are particles with a size ranging from 1 to 1000 nm [2]. 

Even though WWTPs exhibit a notable capacity to eliminate a significant proportion of MPs, with a retention rate ranging from 98% to 99% [3,4,5], they persist as the primary pathway through which MPs reach the environment. This is largely attributable to the massive volume of effluent discharged by WWTPs [6]. Moreover, it is expected that the weathering of macroplastics and MPs will generate secondary NPs, although there is no available data on the environmental loads of NPs [7]. This lack of information regarding the presence of NPs in the environment is especially concerning because studies have reported that nanosized particles primarily induce more toxicity in aquatic and terrestrial animals compared to larger particles [8]. Additionally, investigations have shown that NPs in urban waters differ from MPs in terms of their analytical challenges, transport properties, interactions with pollutants, bio-effects, and removal behavior [9].

In the search for more effective wastewater treatment solutions, membrane-based advanced technologies for wastewater treatment such as membrane bioreactors (MBRs) have emerged as promising competitors. Along with the removal of advanced levels of organic and suspended solid particles, MBRs are also effective in the removal of MPs. MBRs have been demonstrated to reach a removal capacity of 99.9% of MPs (>20 μm) [10]. While MBR systems were neither initially designed for the removal of MPs, their remarkable efficiency in removing them can be attributed to the synergy between the entrapment of MPs in the suspended sludge flocs and the sieving action of the membrane within the reactor [10,11]. 

With the demonstrated effectiveness of MBRs in removing MPs, it is highly important to take one further step in the implementation and assessment of MBR for the removal of NPs from wastewater. Previous studies have shown that 99% of NPs were retained in the sludge during the activated sludge process of a municipal WWTP, highlighting that NPs form aggregates with the suspended solids found in the system [12]. Since MBRs also work with activated sludge, it is reasonable to hypothesize that MBR systems could effectively remove NPs. However, to the knowledge of the authors, no research has been conducted on the impact of NPs in MBR systems thus far. 

Interestingly, the review articles that focused on studies on MBR for MNP removal exclusively cited literature related to MPs [5,13,14]. In the work by Sutrisna et al. [13], a section was dedicated to reviewing studies on the effects of MPs and NPs on MBR. However, a closer look revealed that all of the listed references concentrated solely on the potential use of MBR for the removal of MPs from water and wastewater. Concerning NPs, the review only briefly touches on their biodegradability without addressing MBR efficiency. In fact, Sutrisna et al. [13] concluded that it is crucial to investigate the effects of NPs within the MBR unit, as the presence of these particles could potentially have both positive and negative impacts on MBR operation. On the other hand, MNPs can induce oxidative damage to microbes, disrupt cell membrane integrity, inhibit sludge activity, and reduce the abundance of crucial microbes. This can alter the composition and distribution of extracellular polymeric substances (EPSs) and hinder sludge dewatering, according to the authors of [15]. Alvim et al. [16] studied the influence of 100 nm PS-NPs (10 μg L^−1^) on the microbial community composition and activity of activated sludge and the quality of the final effluent at a sequencing batch reactor (SBR) over 63 days. The authors reported no damage of the activated sludge process under the proposed experimental conditions, meaning that NPs did not modify the global process. However, Zhou et al. [17] found that a high concentration (1000 μg/L) of NPs decreased the nitrogen removal efficiency by activated sludge in the SBR. 

The evaluation of the removal capacity of NPs by advanced water treatment technologies has been limited due to the lack of standardized methods for the sampling, identification, and quantification of NPs from wastewater [15]. The detection and quantification of NPs present unique challenges due to their extremely small size. While techniques such as microscopy, spectroscopy, and dynamic light scattering (DLS) are valuable for qualitative analysis, there remains a need for the improved sensitivity and standardization of methods [18]. Commonly used techniques for MPs include vibrational spectroscopic methods (FTIR or Raman spectroscopy) for the identification and semi-quantification of MPs [19]. These methods can be used to collect appropriate parameters because of the availability of multi-analysis. However, they are limited in that they do not detect particles smaller than 20 µm [20], which is a significant limitation for characterizing and semi-quantifying NPs. 

To overcome these limitations, researchers are exploring the potential of thermal degradation-based technique such as pyrolysis gas chromatography-mass spectrometry (Py-GC-MS), combined with preconcentration strategies that leverage membranes to isolate and enrich NPs from environmental matrices [21]. Membrane technology plays a crucial role in these preconcentration processes by selectively filtering out larger particles and concentrating the smaller NPs, making subsequent analysis more accurate and reliable [22].

In the context outlined above, the present research sought to enrich the understanding of the treatment of wastewater containing PS-NPs using a lab-scale MBR. This study will examine the shift in microbial community composition and the behavior of membrane fouling in an aerobic MBR. The operational behavior of a microfiltration membrane and two ultrafiltration membranes including a recycled one was compared in the presence of NPs. Finally, the semi-quantification of the PS-NPs in both permeates and activated sludge samples were analyzed by Py-GC-MS after their chemical pretreatment. By addressing this critical research gap, our investigation aspires to offer insights into the challenges posed by NPs, contributing to strategies to minimize their environmental impact.

## 2. Materials and Methods

### 2.1. Chemicals

Chemicals used for the preparation of synthetic urban wastewater were glucose ((C_6_H_12_O_6_) D(+) glucose anhydrous, extra pure, Ph Eur, BP, USP), meat peptone, urea (urea reagent grade ACS, sodium chloride (NaCl reagent grade, ACS, ISO, Reag. Ph Eur), sodium bicarbonate (NaHCO_3_, extra pure, Pharmpure^®^, Ph Eur, BP, USP), di-potassium hydrogen phosphate anhydrous (K_2_HPO_4_ for analysis, ExpertQ^®^, ACS, Reag. Ph Eur), calcium chloride dihydrate (CaCl_2_.2H_2_O powder, for analysis, ExpertQ^®^, ACS), and magnesium sulfate heptahydrate (MgSO_4_.7H_2_O for analysis, ExpertQ^®^, ACS, Reag. Ph Eur) supplied by Sigma-Aldrich, Spain. The chemical used for membrane transformation and membrane cleaning was sodium hypochlorite (NaClO 10% *w*/*v*).

The nanoparticles used were PS fluorescent carboxylate with a 116 ± 2 nm size and $\lambda_{ex.}/\lambda_{em.}$: 576 nm/596 nm. These nanoparticles, purchased from IKERLAT Polymers, Spain, are standard, perfectly dispersed, and stable in water.

### 2.2. Membranes

Three flat sheet membranes were investigated in the current study: (i) a microfiltration (MF) membrane (nominal pore size of 0.4 μm), (ii) commercial ultrafiltration membrane UF-PES 150 kDa-Microdyn (c-UF), and (iii) recycled ultrafiltration (r-UF) membrane. It should be noted that all the membrane tests only related to a single run. The membrane characteristics are summarized in Table 1. The nominal molecular weight cut-off (MWCO) values were measured by size exclusion chromatography. The MWCO was defined as the corresponding molecular weight with a retention coefficient of 90%. An Agilent Technologies 1260 Infinity GPC/SEC System with a column from Polymer Labs (PL MIXED aqua gel-OH) with a nominal pore size of 8 μm was used. Milli-Q water was used as the eluent with a 1 mL min^−1^ flow rate. The calibration was carried out with narrow standards of PEOs with molecular weights between 194 and 490,000 Da. The feed solution contained PEOs ranging from 103 to 105 g mol^−1^, keeping the total concentration of 1 g L^−1^ following the composition reported elsewhere [23,24].

The r-UF membranes were obtained by eliminating the polyamide layer of the end-of-life (EoL) reverse osmosis (RO) membranes through exposure to a NaClO dose of 500,000 ppm·h at a pilot scale, following the procedure proposed by García-Pacheco et al. [26]. Furthermore, aligning with the principles of the circular economy [27], the study explored the potential of recycled membranes in MBRs—a field supported by existing research that demonstrates their feasibility and even suggests potential advantages in fouling reduction [28].

### 2.3. Aerobic Membrane Bioreactor (aMBR) System

**Figure 1:**
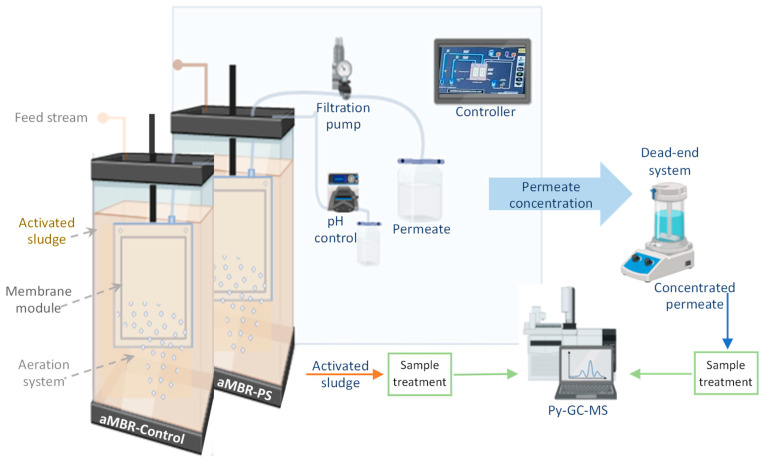
Representation of the experimental setup.

Two systems of submerged-membrane configuration were employed in parallel. The experiments were carried out on a lab scale aMBR at continuous flow. Each aMBR tank had a working volume of 30 L and an effective membrane area (flat sheet membrane module) of 0.11 m^2^. One MBR was used as the control (aMBR-Control) (i.e., without the addition of PS nanospheres), and the other with spiked PS nanospheres (aMBR-PS) (Figure 1). The feed stream used was synthetic wastewater simulating urban wastewater (0.4–0.5 g/L COD), as reported elsewhere [25]. The aMBR feeding was performed by a peristaltic pump (type PPR, SEKO SpA, Barcelona, Spain), and a piston pump FMI (Fluid Metering Inc., Syosset, NY, USA) was used for constant flux operation, whereas a pressure transducer recorded the temporal evolution of the TMP. The reactors were automatically controlled. A pH meter with an integrated temperature sensor (713-type pH meter, Metrohm Ltd., Herisau, Switzerland) was used to monitor the pH and temperature of the bioreactor. The oxygen was supplied by air diffusers positioned at the bottom of the tanks to supply the oxygen required by the biomass and avoid the formation of dead zones by acting like the stirrer system. 

The laboratory-scale aMBR unit was operated at a hydraulic retention time (HRT) of 15 h and the sludge retention time (SRT) was considered infinite since no purge was conducted during the experimental time. Regular measurements of pH, EC, and DO were performed to ascertain that the aMBRs operated under the same conditions. The activated sludge used was taken from the urban WWTP Guadalagua, located in Guadalajara, Spain. Specifically, the samples were collected from the recirculation stream to ensure higher total suspended solid (TSS) concentration values than those from the biological reactor. Prior to starting the experiment, the microbial community was acclimatized for almost 80 days, which ensured that pseudo-steady-state conditions were achieved. The average values remained at 7.86 ± 0.21 pH and 501 ± 0.12 μS/cm in the case of EC. DO concentration in the bioreactor was measured by an oxygen probe (Z921, Consort). A steady-flux value of 18 LMH was employed, whereas the membrane operation was set on cycles of 8 min of suction followed by 2 min of relaxation. The aMBR permeate was analyzed twice a week (i.e., BOD_5_, COD, TN, and TOC) as described in Section 2.4. With the same frequency, the characterization of the mixed liquor properties was conducted (i.e., floc macroscopy by V-30 test, floc microscopy by an optic microscope, and MLSS determination).

The PS nanoparticle (116 ± 2 nm) was chosen as PS has been identified as one of the predominant plastic polymers found in marine environments [7]. Moreover, it is often chosen as a representative for studying the absorption and biotoxicity of MNPs in marine organisms [29]. Thus, the standard (20 g/L) of PS was added directly to the mixed liquor (i.e., NPs were added manually to the reactor to prevent their loss through the pump and tubing system). The addition was conducted daily during the first two weeks to reach a 20 mg/L concentration in the reactor (stage I). Thereafter, the addition was stopped for one week. Later, from day 21., the concentration of spiked PS was increased for the following week (stage II) to assess the eventual effects of an abrupt increase in NP concentration (up to 60 mg/L) on the MBR system. Finally, the MBR was operated under the total accumulated concentration from the two spiked stages for 5 more weeks without any other PS addition to evaluate the performance of different membranes under the same concentration of NPs. The MF membrane was used for the initial 39-day experiment. Following this, UF membranes (i.e., c-UF and r-UF) were tested individually for two weeks each.

### 2.4. Permeate and Sludge Characterization

#### 2.4.1. Analytical Methods for Wastewater Parameters

The main parameters for wastewater analysis were measured according to standard methods [30] that included EC, pH, BOD_5_, COD, TOC, TN, and MLSS. The conductivity values of the solutions were measured by a CM 35 conductivity meter (Crison Instrument, Barcelona, Spain). TOC was measured by a TOC analyzer (TOC-5000A, Shimadzu Co., Kyoto, Japan). TN was determined colorimetrically (UV-1700 Spectrophotometer, Shimadzu Co.) after sample digestion using sulfuric acid/peroxodisulfate and alkaline potassium persulfate, respectively. MLSS was measured by filtration on a Whatman GF/A microfiber glass filter (1.6 μm nominal pore size). The sludge volumetric index (SVI) was calculated following Equation (1).

(1) $$SVI=\frac{V_{30}\left[\frac{ml}{L}\right]\;*{\;2}^{\left(n\right)}}{MLSS\left[\frac{g}{L}\right]}$$

where $V_{30}$ is the volume of settled mixed liquor measured at 30 min and n is the dilution number of the mixed liquor. 

Data were analyzed statistically using a statistical significance level of 0.05 by *t*-test analysis.

#### 2.4.2. Microbial Community Analysis and SMP

DNA from the MBR samples was extracted using a FastDNA SPIN^®^ Kit for soil (MP Biomedicals, Madrid, Spain), according to the protocol from the manufacturer (FastDNATM Spin Kit for Soil DNA Extraction|MP Biomedicals, n.d.) [31]. DNA from all samples was extracted twice, obtaining 100 µL as the final volume. Inhibitors in the sample were removed by employing a One-Step PCR Inhibitor Removal Kit (Zymo Research, Irvine, CA, USA). DNA concentration was measured using the Qubit^®^ dsDNA BR Assay Kit (Molecular probes, Eugene, OR, USA), and the absorbance rate (260/280 nm) was measured with a NanoDrop ND-1000 UV/Vis spectrophotometer (Thermo Fisher Scientific, Waltham, MA, USA). DNA samples were sequenced for V4 16S rRNA gene amplification using the primers 515F–806R. The subsequent amplicon sequencing was conducted on the Illumina Miseq platform.

To obtain the SMP in terms of carbohydrates and proteins, the mixed liquor samples were centrifuged at 15,000 rpm for 10 min at 4 °C. Later, the liquid part was filtered with a 0.45 µm filter size of cellulose acetate to obtain the soluble portion. To determine the carbohydrate concentration, the anthrone method [32] was employed. Protein concentrations were determined following the bicinchoninic acid (BCA) method [33].

#### 2.4.3. Semi-Quantification of PS Nanoplastics by Pyrolysis Gas Chromatography-Mass Spectrometry (Py-GC-MS)

##### Sampling and Sampling Treatment

Activated sludge samples were collected once per week. To ensure representative sampling of the activated sludge, a sampler was utilized to take samples from the middle of the reactors. A volume of 10 mL of mixed liquor was treated prior to the analysis by applying a peroxidation process. The peroxidation reaction was conducted with 10 mL of the sample (mixed liquor) and 20 mL of peroxide (H_2_O_2_, 30% wt) for 4 h at a temperature of 60 °C, following the methodology outlined in a previous study by Bretas Alvim et al. [34]. Subsequently, the digested sample was vacuum filtered using alumina filters (Anodisc^TM^ 47 mm, 0.02 µm), and the filter was then dried at 50 °C in a laboratory oven for 1 h. Finally, the dried filter was milled in a ball mill for two minutes at 30 Hz and an aliquot of 1 mg was taken for Py-GC-MS analysis.

Permeate samples were also collected once per week. The analyzed volume of the different permeate samples ranged from 100 to 500 mL, which had been pre-concentrated from an initial volume of 5 to 8 L. The pre-concentration process was carried out in a dead-end system with regenerated cellulose (RC, 30 kDa) membranes, as shown in Figure 1. The RC membrane was chosen due to the results obtained in previous studies [22] that showed fouling remotion and permeability recovery values up to 100%. The pre-concentrated permeate samples were also chemically digested by peroxidation reactions in a ratio of 100:1 (permeate sample: H_2_O_2_, 30% wt) for 2 h at a temperature of 60 °C following the same methodology by Bretas Alvim et al. [34]. The subsequent steps after digesting the sample were identical to those described for the mixed liquor (vacuum filtering, drying, and milling). 

##### Identification and Semi-Quantification

A pyrolyzer 6200 (CDS Analytical) coupled to a gas chromatography-mass spectrometer (7890B and 5977B MSD, Agilent Technologies) was used for plastic identification and semi-quantification. Pyrolysis was performed at a temperature from 150 to 600 °C (1 min). The transfer line until the gas chromatograph was maintained at 300 °C. The GC injector operated in split mode with a 100:1 ratio at a temperature of 300 °C. Pyrolysis products were separated using He (1.5 mL/min) as the carrier gas in the HP-5 capillary column (30 m length × 0.32 mm i.d. × 0.25 µm film thickness; Agilent). The GC oven program was as follows: 2 min at 40 °C, then increased up to 325 °C at 10 °C/min and held for 2 min. The GC-MS interface temperature was fixed at 280 °C. The mass spectrometer operated under electron ionization mode (70 eV); the source temperature was kept at 230 °C. The acquisition was performed under scan mode ranging from 40 to 400 *m*/*z*. The pyrolytic products used as indicators of the presence of PS are indicated in Table 2 including the most specific *m*/*z* ions selected for each compound. 

Although it is known that styrene can be generated from other sources, it was selected as the quantifying peak in this study as the recoveries in the real samples were satisfactory (see Supplementary Materials S2).

The quantification of polymers was conducted by injecting calibration curves using specific standards. The description of the preparation of the standards and validation of the method is given in Supplementary Materials S2. The limit of detection and quantification (LOD and LOQ) were defined according to the matrix (activated sludge and permeate) and the volume treated, also referred to in Supplementary Materials S2.

The treatment and analysis of both types of samples were conducted under strict protocols to avoid cross-contamination and altered results (see Supplementary Materials S1).

### 2.5. Membrane Characterization

#### 2.5.1. RIS Analysis

To understand the fouling mechanism during the studied processes, the resistance-in-series (RIS) analysis was realized following the model proposed by [35]:

(2) $$R_{t}=\frac{TMP}{J\;\mu }$$

where J is the permeate flux of the fouled membrane [m^3^ m^−2^ s^−1^], TMP is the transmembrane pressure [Pa], and μ is the permeate viscosity [Pa s] at the operating temperature. By the end of the studied period, the membranes were removed from the bioreactor and physically cleaned by rinsing with tap water and soft mechanical cleaning following the procedure of Rodríguez-Sáez et al. [25]. The cleaned membrane was then immersed in clean water and exposed to the same operational filtration conditions as during the experiment in order to measure the resistance to filtration in clean water ($R_{t,cw}$):

(3) $$R_{t,cw}=\frac{{TMP}_{cw}}{J_{cw}\;\mu }$$

where $J_{cw}$ is the permeate flux of the cleaned membrane [m^3^ m^−2^ s^−1^] measured with clean water, ${TMP}_{cw}$ is the transmembrane pressure [Pa], and μ is the permeate viscosity [Pa s] at the operating temperature. 

The total resistance is expressed as follows in Equation (4):

(4) $$R_{t}=R_{m}+R_{pb}+R_{c}$$

where $R_{m}$, $R_{pb}$, and $R_{c}$ represent the membrane, the pore blocking, and cake layer resistance contributions, respectively. The total resistance $R_{t,cw}$ during clean water operation can be expressed as (Equation (5)):

(5) $$R_{t,cw}=R_{m}+R_{pb}$$

Fouling resistance ($R_{f}$) is defined as the total fouling resistance excluding the membrane resistance $R_{m}$ (Equation (6)):

(6) $$R_{f}=R_{c}+R_{pb}$$

#### 2.5.2. Scanning Electron Microscopy (SEM) 

Scanning electron microscopy (SEM) using the S-8000 Model (Hitachi) image device was employed to observe the morphology of the membrane surfaces and the fouling degree of the studied membranes after the physical cleaning of the membranes at the end of the experiment. Preceding the microscopy analysis, the membrane samples were dried by heating at 50 °C for 48 h.

#### 2.5.3. Confocal Laser Scanning Microscopy (CLSM)

The membranes were also observed under a confocal laser scanning microscope (CLSM Leica SP5, Leica Microsystems). Each membrane was cut into pieces of 5 mm × 5 mm (i.e., the membrane from the MBR-Control system and the other from the MBR-PS system) and three different areas were examined. The resulting images were analyzed by ImageJ software (Version 1.51n). The 3D projection images of the particles were constructed by the ImageJ 3Dviewer plugin. 

## 3. Results

### 3.1. Activated Sludge

#### 3.1.1. Changes to Settleability of Sludge and SMP

MLSS and SVI were measured periodically to characterize the settleability capacity of the sludge. SVI values were calculated with n = 3 (i.e., a dilution 1:3 of mixed liquor: permeate water) to obtain representative values (i.e., values below 400 mL/g MLSS). The SVI decreased from 218.3 mL/g MLSS (day 1) to 199.2 mL/g MLSS (day 39) in the MBR-Control, while the SVI in the MBR-PS decreased from 206.2 mL/g MLSS (day 1) to 202.9 mL/g MLSS (day 39). Even though the variation in SVI in MBR-PS was <2% compared to <9% in the MBR-Control, which might have been influenced by the plastic mass accumulation in the reactor, the results showed that the presence of polystyrene nanospheres in the mixed liquor did not significantly affect the settleability in terms of the volumetric index. This is in concordance with results obtained for the low concentration of nano-sized PS (213.7 ± 1.7 nm) by Xu et al. [36]. However, physical changes such as a variation in the color were observed since the employed PS nanospheres had a fluorescent dye incorporated in the particles. The color of the sludge changed from brown to reddish, which may indicate the absorption of NPs to the activated sludge flocs. Li et al. [37] studied the effect of short-term exposure to polystyrene nanoparticles on activated sludge performance. They also observed a color shift of the activated sludge from dark brown to light yellow and attributed it to the adsorption of the white PS-NPs by the activated sludge. 

Soluble microbial products (SMPs) are the most direct indicator relating to membrane fouling [38]. SMPs are also reported in the literature as soluble extracellular polymeric substances (S-EPSs) and mainly consist in the portion of EPSs that are not associated with the microbial cell but are solubilized in the mixed liquor [39]. SMPs are related to cell lysis, substrate metabolism, and biomass growth [40], which can be affected by many influencing factors such as the substrate type, nutrient content, solid retention time, presence of toxic substances, the shear rate of a reactor, and salinity [41]. The major components of SMPs are carbohydrates (SMPc) and proteins (SMPp) [42]. Therefore, the SMP concentration, expressed as SMPc and SMPp, were determined at three different moments of the experiment: (1) at the beginning, (2) at the end of the first period of PS addition, and (3) at the end of the experiment with the MF membrane (day 39).

In the first stage of the experiment (i.e., up to 20 mg PS L^−1^), the obtained concentrations of SMPc were 1.54 mg L^−1^ and 1.15 mg L^−1^ while the SMPp values were 4.06 mg L^−1^ and 5.58 mg L^−1^ in the MBR-Control and MBR-PS, respectively. The values obtained in both the MBR-Control and MBR-PS reactors showed no significant difference in the average SMP values, which means that the presence of PS nanospheres at a low concentration did not provoke any biomass disruption. Previous reports have shown the toxicity of NPs as a dose-dependent manner, where low concentrations of PS-NPs (0.1, 1, 5, 10, and 50 mg L^−1^) did not exhibit obvious toxicity [43,44]. However, even though the levels of SMPs observed in the biomass flocs during the course of this study were relatively low, at the end of the 39-day experiment, when the accumulation of PS-NPs was higher (i.e., around 60 mg L^−1^), the behavior changed. The most significant variation was observed in the carbohydrate measurements. The increase in the SMPc values in the MBR-PS was double compared with the control. While the MBR-Control increased more than 2.5 times, the MBR-PS showed more than five times the SMPc increment, most probably due to the protective responses of bacteria under nanoplastic stress. A similar behavior was observed by Tang et al. [45], who studied the influence of three different PS nanoplastic concentrations (PS-0.1, PS-1, and PS-10 mg L^−1^) on the S-EPS variation [45]. They observed an increase in S-EPSs in all of the studied reactors after exposure to low doses of PS-NPs. They concluded that the production of S-EPSs on the bacteria cell surfaces helped to protect them from harm when they exposed to PS-NPs [45]. 

Regarding the protein concentrations, they remained almost constant in both cases, obtaining values of 3.99 mg L^−1^ and 4.93 mg L^−1^ in the MBR-Control and MBR-PS, respectively. Wang et al. [29] observed that a relatively lower concentration (0.14–0.30 g L^−1^) of polypropylene microspheres (500 µm) in an 84-day experiment stimulated the release of SMP [46]. Similarly, the content of S-EPS was increased slightly after PS-NP (100 mg L^−1^) exposure in the study conducted by Qian et al. [47], which agrees with the results in the current study. 

#### 3.1.2. Microbial Community Analysis

Microbial communities were examined using microscopy observations and metagenomic analysis. The initial conditions of the experiment were similar for both reactors under investigation, where the predominant bacteria observed in both reactors were identified as morphotypes 0803/0914 and 0092. These morphotypes are associated with the phylum Chloroflexi. This finding aligns with the expected abundance of Chloroflexi in urban WWTPs, from which the utilized sludge was obtained. During the subsequent sample observations (i.e., intermediate sample), the relative abundance of the initial morphotypes remained, along with the presence of type 1851, which also appeared in the Chloroflexi phylum. However, morphotype 0092 was observed only in the control reactor. At the conclusion of the experiment, the bacterial community in the MBR-Control maintained was similar to that at the intermediate sample. In contrast, in the MBR-PS, there was a noticeable change in the bacterial community, specifically with the observation of morphotype 0041/0675, replacing the previous observation of morphotype 1851. 

**Figure 2:**
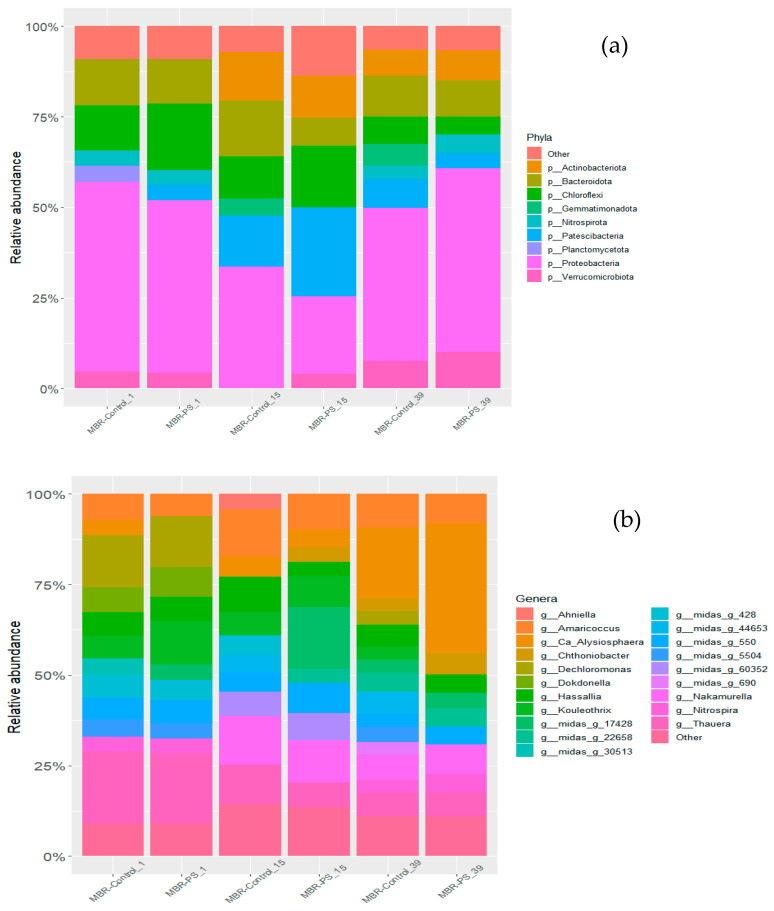
Bacterial community composition at the phylum level (**a**) and genera level (**b**) in both reactors (i.e., MBR-Control and MBR-PS) sampled on the initial day (day 1st), on day 15, and day 39.

16 S rRNA gene sequencing was also investigated (1) at the beginning, (2) at the end of the first period of PS addition, and (3) at the 39th day. Bacterial communities were dominated by Proteobacteria in both reactors (average of 30.99% of 16S rRNA gene reads) during the study. This result is in agreement with Rehman et al. [48], who studied the composition and functional potential of the microbial communities of a lab-scale MBR system with a submerged ultrafiltration (UF) membrane configuration. As Figure 2 shows, other major groups were the Planctomycetota (15.11%), Bacteroidota (13.45%), Chloroflexi (8.61%), Verrucomicrobiota (6.33%), Patescibacteria (5.67%), Actinobacteriota (4.24%), Actinobacteriota (4.24%), Acidobacteriota (3.07%), Myxococcota (2.42%), Gemmatimonadota (1.45%), Nitrospirota (1.44%), and Bdellovibrionota (1.42%) (Figure 2a). Previous studies have also observed the same phyla predominance in conventional activated sludge reactors [49,50].

**Figure 3:**
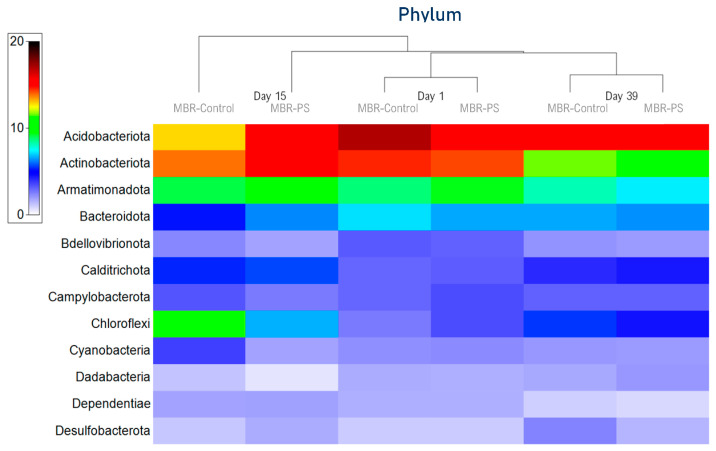
Heat-map of the bacterial community composition at the phylum level with cluster analysis. The color intensity in each panel shows the percentage in a sample, referring to the color key at the left.

Minor changes were observed with the most abundant phylum Proteobacteria along with Planctomycetota, and Verrucomicrobiota. At the same intermediate sample, the relative abundance of Proteobacteria dropped from 31% to 25%, from 15% to 13% in the case of Planctomycetota, and from 7% to 5% for Verrucomicrobiota. In the last sample analyzed (day 39), all phylum recovered the relative abundance of the initial values obtained in the control reactor. Furthermore, the results shown in Figure 2 suggest that the presence of PS at low concentrations (first stage of treatment) and for a short period have more effect on the bacterial community than higher concentrations for a longer time. In the cluster analysis of Figure 3, the adaptation capability of the medium to the pollutant presence over time was more noticeable.

The relative abundance of Patescibacteria increased from 3% to 7% and 11% in the MBR-Control and MBR-PS, respectively, when the PS remained at low concentrations (stage I). Alvim et al. [51] recently studied the effect of PS 100 nm size on the activated sludge in a sequencing batch reactor. The same results were found with the Patescibacteria phylum at low concentrations of nanoplastics. Other studies have also agreed with the enrichment of Patescibacteria in the presence of plastic [45] and suggested this phylum as a possible bacterial biomarker for plastic contamination [51]. Even though there are similar findings in terms of the abundance of some bacterial communities in the presence of NPs, a direct comparison between the conventional activated sludge (CAS) and MBR system behavior is not straightforward. This is due to the relatively longer operational time and higher MLSS concentration associated with MBR technology compared to CAS [52]. Consequently, the cumulative impact of nanoplastics could diverge from conventional sludge technologies, potentially leading to variations in bacterial relative abundance fluctuations. In our study, at the end of the second cycle of PS addition, the relative abundance of Patescibacteria decreased again up to 5% in both reactors, while the accumulation and homogeneity of the nanoplastic in the MBR increased. Given the intermittent addition of PS, the results suggest that the bacterial community is capable of adapting, following a period of rest. 

### 3.2. Membrane Operation

#### 3.2.1. Permeate Quality 

The experiment (once the addition of PS started) was conducted with an MF membrane (nominal diameter of 0.4 µm) for 39 days, followed by testing the UF membranes including c-UF and r-UF for two weeks for each one. Table 3 summarizes the characteristics of the effluents of both MBRs, where the results are expressed in average values and their respective standard deviations (±SD). 

In general terms, the effluents from both the MBR-Control and MBR-PS reactors were very similar in terms of characteristics. A percentage of COD removal above 97% was obtained in both reactors (MBR-Control and MBR-PS) for all membranes, which represents a high purification capacity. Results showed that the presence of nanoplastics (PS) did not affect the remediation capacity of the MBR for organic matter. Similar high removal efficiencies (effluent COD: 20.2  ±  10.2–23.8  ±  13.4 mg L^−1^) were previously achieved by Maliwan et al. [52] during their study of different MP additions (0, 7, 15, and 75 MPs/L, respectively). Furthermore, other studies related to biological wastewater treatment have reported COD removal efficiencies of sequencing batch reactor (SBR) during an experimental time above 90% [16], and 97.35 ± 0.81% (n = 33) of COD removal was obtained in SBR-MP, representing a high purification capacity of the reactor in the presence of polyethylene microbeads [53]. It is also worth noticing that the r-UF showed a higher removal efficiency compared to commercial ones, despite the results summarized in Table 3. The chlorination transformation process modified key surface characteristics [54], particularly achieving a MWCO of 30,000 Da, which could influence the observed COD removal efficiency.

The quality of permeate streams from samples obtained from the three types of membranes (i.e., MF, r-UF, and c-UF) was also verified in terms of PS presence. Before conducting the Pyr-GC-MS analysis, a pre-concentration step was carried out to enhance the detectability of trace nanoplastics. The pre-concentrated permeate samples were analyzed from a larger initial volume from 5 to 8 L, as explained in Section 2.4.3. According to the results, no presence of PS was detected, since all values were below the limit of detection (LOD: ranging from 0.013 to 0.067 µg mL^−1^). Consequently, the rejection capacity of the MBR toward PS nanoplastics was 100% with all of the studied membranes. 

In addition, the verification of PS concentration in sludge was conducted weekly. Since the comparison study of membrane operation occurred sequentially (i.e., r-UF following c-UF), four measurements using Py-GC-MS were controlled. Each time, the Py-GC-MS analyses were performed on both types of samples (i.e., from MBR-Control and MBR-PS) to confirm the presence of PS in the sludge.

As could be verified, the PS concentration was kept almost constant (Table 4) during the comparative study. Accordingly, Mitrano et al. [12] reported that PS-NPs have the propensity to rapidly transition from the aqueous phase to the sludge phase within a short timeframe. Consequently, the swift formation of the cake layer and the strong adsorption capability of activated sludge for PS-NPs can act as a physical barrier, hindering the movement of additional nanoparticles and limiting their ability to contribute significantly to pore blocking.

#### 3.2.2. Transmembrane Pressure (TMP) Evolution

**Figure 4:**
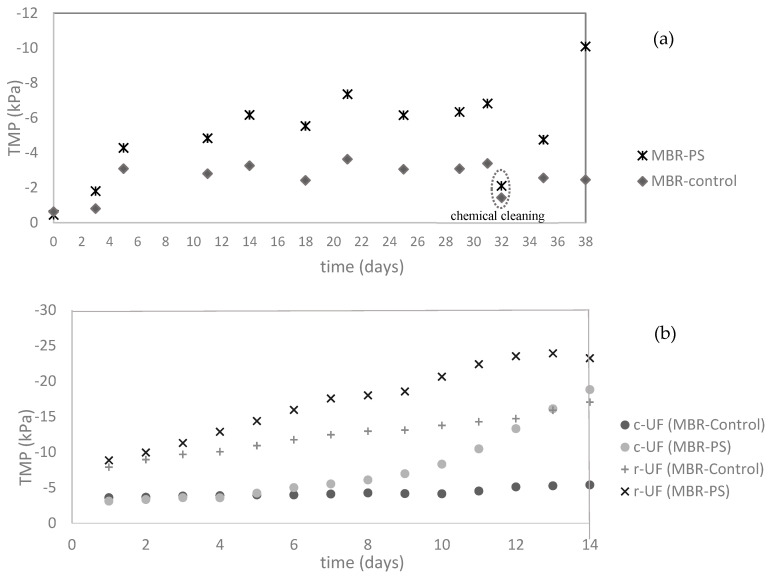
Variation of the transmembrane pressure (TMP) over the experimental time of the (**a**) microfiltration membrane (the highlighted TMP values on the 32nd day relate to chemical cleaning of the membranes) and (**b**) ultrafiltration membranes (comparison between r-UF and c-UF).

The effect of the presence of PS was noticeable in the transmembrane pressure (TMP) increment. Figure 4 shows the TMP evolution over the experimental time for the three studied membranes.

As can be observed in Figure 4, the overall increase in TMP in the MBR-PS with the increment of PS addition was higher than in the MBR-Control (i.e., no presence of PS) for the three membranes employed. This indicates that nanoplastics contribute significantly to membrane fouling over time.

For the case of the MF membrane (Figure 4a), throughout the experimental period, regular physical cleaning of the membranes was carried out weekly, along with a single chemical clean at the conclusion of polystyrene (PS) addition (day 32). It is important to note that the inflection points in Figure 4a correspond to the instances of membrane cleaning. Finally, the system was kept running without PS addition for another week. Toward the end of the evaluated time, while the TMP in the MBR-Control increased less than two times, the growth of the TMP in the MBR-PS was almost five times higher than the initial TMP. This behavior shows the interactions between NPs and the membrane, which enhanced the membrane fouling and consequently the overall process performance of the wastewater treatment by MBR. 

Subsequent to the 39-day period of the experiment, a comparative study of the UF membranes (c-UF and r-Uf) was carried out. The membrane fouling of c-UF and r-UF induced by the presence/absence of PS was studied, with the results given in Figure 4b. It was observed in Figure 4b that while r-UF exhibited a lineal (R^2^ > 0.97 for both r-UF membranes) TMP increase, with the TMP of the membrane from MBR-PS higher than that from the MBR-Control, the TMP of the c-UF membrane behaved differently. After day 8, the c-UF submerged in MBR-PS presented a sharp TMP increase. On the other hand, the r-UF submerged in MBR-PS tended to stabilize. This stabilization is a promising result and could be attributed to the NaClO treatment used in the recycling process, which modifies the membrane-surface properties, potentially reducing its susceptibility to biofouling [25,28].

#### 3.2.3. RIS Analysis and Surface Characterizations

**Figure 5:**
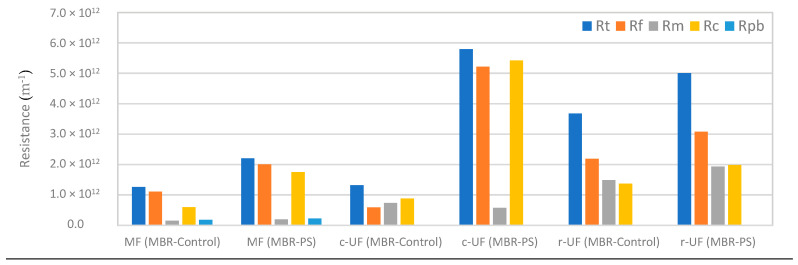
Resistances of the MF, commercial UF (c-UF), and recycled UF (r-UF) membranes in the control (MBR-Control) and the experimental (MBR-PS) reactors. $R_{t}$: total resistance; $R_{f}$: fouling resistance; $R_{m}$: membrane resistance; $R_{c}$: cake layer resistance; $R_{pb}$: pore blocking resistance.

Figure 5 reports the RIS model outputs of the three membranes: MF, c-UF, and r-UF. The presented $R_{c}$ values include both reversible and irreversible cake layer resistance, whereas the $R_{f}$ values comprise the resistances of the cake layer and the pore-blocking mechanism.

The major contribution to the total resistance was from the cake layer resistance (Rc) across all three membrane types assessed (MF, c-UF, and r-UF), as depicted in Figure 5. 

For the case of MF (0.4 μm), one might have hypothesized a significant increase in the pore blocking resistance (Rpb) of the membrane due to the accumulation of the nanospheres (116 nm) inside the membrane. However, the primary contribution to the overall resistance originated from Rc (cake layer formation). This suggests that even with larger pores, the formation of a cake layer by SMPs (explained in Section 3.1.1) and potential biofilm can significantly impede the flow. The major SMP release is directly linked to the formation of the cake layer. During filtration, SMPs are believed to adsorb onto the membrane surface, blocking membrane pores and/or forming a gel layer on the surface. This process provides a potential nutrient source for biofilm formation, thereby increasing the resistance to permeate flow [39].

On the other hand, it is important to note that the Rm of the r-UF membrane was the highest value among the compared membranes, as depicted in Figure 5. This difference can be attributed to the intrinsic properties of the recycled membranes, which experience a manufacturing process (transformation from EoL to r-UF) involving the removal of the first layer and the emergence of the polyethersulfone layer after use. Additionally, the r-UF membrane had the smallest pore size, as Table 1 depicts, which resulted in a higher resistance to water permeation. However, a close examination of the fouling resistance behavior revealed a tendency of r-UF toward lower values of Rf than those of the c-UF membrane (as shown in Figure 5). This reinforces the previous observation of potential fouling reduction benefits associated with recycled UF membranes and aligns with the TMP evolution explained in the previous section. Thus, the application of r-UF membranes in a flat sheet configuration within an aMBR system for both the absence and presence of NPs demonstrates promising results.

#### 3.2.4. Membrane Characterization

**Figure 6:**
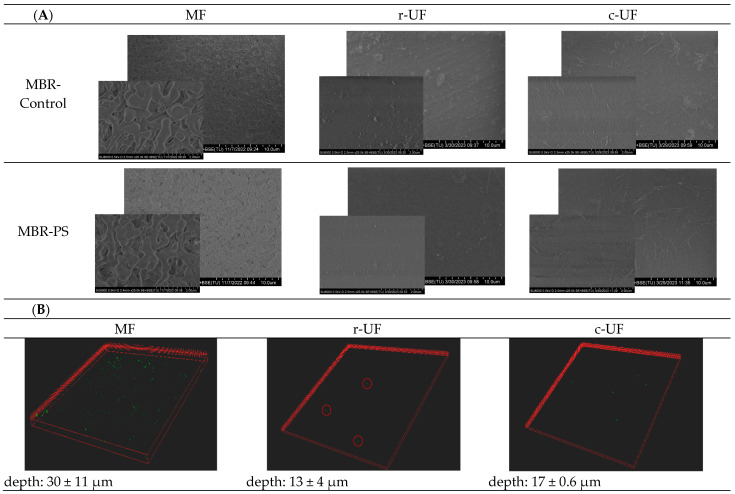
(**A**) Membrane surface micrographs of the membranes at the end of the experiment after the physical clean: membranes from the MBR-Control (second row) and membranes from MBR-PS (third row). (**B**) 3D projection from confocal laser scanning microscopy of the MBR-PS membranes. Green dots in the 3D projection of the membranes represent the PS nanoparticles.

To support the previous observations, two different techniques after the physical clean at the end of each assessment were employed. Figure 6 shows the SEM micrographs of the MF and UF membranes (A) and the three-dimension (3D) projections from the confocal micrograph (B).

SEM images of the studied membrane surfaces did not reveal any visible evidence of PS deposition or attachment. To obtain a more comprehensive understanding of potential PS fouling, confocal microscopy was employed as a complementary technique. Unlike SEM, 3D imaging confocal microscopy allows for depth profiling, which can reveal PS particles that may have penetrated the membrane structure.

Confocal images from the MBR-Control samples were not presented because no fluorescent particles were observed, resulting in entirely black images devoid of fluorescent dots. This absence of fluorescent signals in the control samples served as the baseline, confirming that any fluorescent dots observed in other samples can be attributed to PS particles. A 3D projection from the confocal microscope showed the portion of infiltrated nanoplastics adhered within the pores of the MF membrane. This aligns with prior studies by Abdelrasoul et al. [55], who reported the fouling effect of small particles falling within the critical size range, which is defined as the size range between 1/6 and 1/2 of the pore diameter. This fouling leads to particle attachments in the internal wall of the membrane pores, decreasing the membrane effective pore size [55]. However, in the current study, the portion of nanoplastics that infiltrated was very small, and consequently did not significantly contribute to the internal pore blocking. For the case of the UF membranes, the CLSM images exhibited a significantly lower attachment of PS nanospheres on their surfaces after the physical cleaning protocol was conducted. 

The analysis of the 3D projection from CLSM provides insights into the depth of PS penetration into the different membrane structures (Figure 6B). For MF membranes, the depth of the PS-containing layers was around 30 μm. In contrast, the depth of the PS layers was approximately half for the UF membranes. This difference revealed the more effective sieving capability of both UF membranes in preventing the passage of PS nanoplastics compared to the MF membrane. 

Nevertheless, it is worth noting that the fouling contribution of the presence of PS may vary over time, influenced by potential interactions between the nanoparticles and their accumulation within the membrane. Further research should be conducted to investigate the long-term effects of these NPs. 

## 4. Conclusions

This study demonstrates that aerobic membrane bioreactors are highly effective in removing nanoparticles from wastewater, achieving over 97% COD removal in both the control and PS-exposed reactors. However, the presence of nano-sized PS particles negatively affected the membrane performance, enhancing the fouling phenomena and increasing the transmembrane pressure. This impact was particularly evident in changes in the soluble microbial products, especially carbohydrates, which are key contributors to fouling. The recycled UF membrane showed lower fouling tendencies than the commercial UF membranes, highlighting the potential benefits of surface modifications. Despite these challenges, aerobic MBRs remain a promising solution for NP removal, though membrane fouling requires further attention.

## Figures and Tables

**Table 1:** Technical data of the studied membranes.

	Manufacturer			
Membranes	Material	Nominal Pore Size	Nominal MWCO (Da)	Nominal MWCO (Da)
MF	Chlorinated polyethylene	0.4 µm	---	---
r-UF	Polyethersulfone	12 nm [25]	---	30,000
c-UF	Polyethersulfone	<30 nm	150,000	90,000

**Table 2:** Characteristic pyrolytic products obtained for polystyrene and *m*/*z* ions selected as indicators for each peak.

Polymer	Pyrolytic Products	Indicator Ions (*m*/*z*)
PS	Styrene *	104, 78, 51
	3-Butene-1,3-diyldibenzene (Styrene dimer)	91, 104, 130
	5-Hexene-1,3,5-triyltribenzene (Styrene trimer)	91, 117, 194

* Compound used for quantification.

**Table 3:** Average effluent quality and removal capacity of MBR.

	pH	CE (µS/cm)	COD (mg/L)	%R (COD)	TOC (mg/L)	%R (TOC)	Total N (mg/L)	%R (TN)
	**MF**							
**MBR-** **Control**	7.90 ± 0.20	564.92 ± 0.17	10.28 ± 2.30	97.44 ± 1.82	2.28 ± 0.65	97.88 ± 2.00	15.63 ± 4.50	49.60 ± 14.52
**MBR-PS**	7.91 ± 0.12	518.08 ± 0.17	8.84 ± 3.05	97.80 ± 0.76	1.83 ± 0.61	98.38 ± 0.84	18.75 ± 3.45	39.52 ± 11.14
	**c-UF**							
**MBR-** **Control**	7.91 ± 0.10	482.60 ± 0.10	8.48 ± 1.39	97.89 ± 0.35	2.05 ± 0.82	98.46 ± 0.62	12.75 ± 2.50	50.96 ± 9.62
**MBR-PS**	7.93 ± 0.12	452.80 ± 0.08	9.18 ± 0.59	97.71 ± 0.15	1.98 ± 0.33	98.51 ± 0.25	18.00 ± 2.65	30.77 ± 10.18
	**r-UF**							
**MBR-** **Control**	7.80 ± 0.08	440.75 ± 0.02	5.33 ± 0.67	98.61 ± 0.17	1.19 ± 0.12	99.09 ± 0.10	14.55 ± 1.67	46.18 ± 4.64
**MBR-PS**	7.92 ± 0.08	516.25 ± 0.09	5.88 ± 1.17	98.47 ± 0.34	1.07 ± 0.27	99.18 ± 0.21	18.25 ± 4.11	32.01 ± 17.18

**Table 4:** Summary of the PS quantification in sludge by Py-GC/MS.

	*c-UF Membrane*		*r-UF Membrane*	
	Initial Point	Second Week	Third Week	Fourth Week
*Conc. PS (mg L^−1^)*	52.86	52.31	55.03	55.27

## Data Availability

The original contributions presented in the study are included in the article, further inquiries can be directed to the corresponding authors.

## Supplementary Materials

The following supporting information can be downloaded at: https://www.mdpi.com/article/10.3390/membranes14080174/s1, Figure S1: Calibration curve for PS from 0.1 to 5 µg; Table S1: Preparation calibration curve; Table S2: Instrumental LOQ and LOD established for each matrix.

## Abbreviations

UF	Ultrafiltration	µ	Dynamic viscosity (Pa·s)
c-UF	Commercial ultrafiltration	J	Flux (L·m^−2·^h^−1^)
r-UF	Recycled ultrafiltration	Rt	Total resistance (m^−1^)
MF	Microfiltration	Rc	Cake layer resistance (m^−1^)
aMBR	Aerobic membrane bioreactor	Rf	Fouling resistance (m^−1^)
MNPs	Micronanoplastics	Rpb	Pore blocking resistance (m^−1^)
MPs	Microplastics	Rwt	Total resistance measured with clean water after physical cleaning (m^−1^)
NPs	Nanoplastics	Rm	Membrane resistance (m^−1^)
PS	polystyrene	NaOCl	Sodium hypochlorite
RO	Reverse osmosis	COD	Chemical oxygen demand
PA	Polyamide	TSS	Total suspended solid
TMP	Transmembrane pressure (Pa)	D	Diameter
WWTPs	Wastewater treatment plants	PR	Permeability recovery
SBR	Sequencing batch reactor	±SD	Standard deviations
SRT	Sludge retention time	EPS	Extracellular polymeric substances
HRT	Hydraulic retention time	DLS	Dynamic light scattering
SEM	Scanning electron microscopy	Pyr-GC-MS	Pyrolysis gas chromatography-mass spectrometry
CLSM	Confocal laser scanning microscopy		
SEC	Size Exclusion Chromatography		
MWCO	Molecular weight cut-off		
