# Supplementary material for: Treatment of Synthetic Wastewater Containing Polystyrene (PS) Nanoplastics by Membrane Bioreactor (MBR): Study of the Effects on Microbial Community and Membrane Fouling
Source: Membranes (Basel). 2024 Aug 9;14(8):174. doi: 10.3390/membranes14080174 (PMC11356002; doi:10.3390/membranes14080174)
Supplement: Supplementary file 1 [file membranes-14-00174-s001.zip › membranes-3120811-supplementary.pdf]

Supplementary Material

for:

Article

Treatment of synthetic wastewater containing polystyrene (PS) nanoplastics by Membrane Bioreactor (MBR): study of the effects on microbial community and membrane fouling

Anamary Pompa-Pernía[1,2,*], Serena Molina[1], Laura Cherta[1], Lorena Martínez-García[1], Junkal Landaburu-Aguirre[1]

[1] IMDEA Water Institute, Avenida Punto Com, 2, Alcalá de Henares, 28805 Madrid, Spain

[2] Chemical Engineering Department, University of Alcalá, Ctra. Madrid-Barcelona Km 33.600, Alcalá de Henares, 28871 Madrid, Spain

* Correspondence: anamary.pompa@imdea.org

S1. Quality Control

To ensure the reliability and accuracy of our experimental setup, a series of quality control measures were implemented during the collection, processing, and analysis of samples.

- **Laboratory Attire and Hygiene:**

Laboratory personnel wore cotton lab coats to minimize the risk of introducing external contaminants. Gloves were worn throughout the sample processing procedures to prevent contamination from skin contact.

- **Sample Handling:**

During filtration, a glass cover was placed on top of the filtration unit to shield the samples from airborne particles. Prior to use, all containers used for sample processing were cleaned with distilled water.

- **Process Blanks:**

A process blank was prepared by replicating all steps of sample digestion, filtration, and storage without actual sample material. This blank was processed in parallel with the samples to monitor for any potential contamination introduced during the experimental procedures. The inclusion of process blanks enabled the identification and correction of any contamination sources, ensuring the integrity of the experimental results.

- **Cross-Contamination Prevention during Analysis:**

During Py-GC-MS analysis, blank runs were conducted between each sample analysis. These blank runs were essential to detect and eliminate any residual contamination from previous samples, thereby preventing cross-contamination and ensuring the accuracy of subsequent measurements.

S2. Preparation of Standards and Instrumental Validation Py-GC-MS

Preparation of Standards and the Calibration Curve

PS dissolved in THF was used as standard for the quantification with the Py-GC-MS. 20 mg of PS was dissolved in 1 mL of THF using ultrasound for 20 minutes and the resulting solution of 20 mg mL^{-1} was used for the preparation of the calibration stocks by dilution. From the stocks of 2000, 200 and 20 mg mL^{-1}, different volumes were taken to prepare the different calibration points directly into the pyrolysis tubes. Before injecting them into the system, the solvent was allowed to evaporate. The specific conditions to prepare the six calibration points (from 0.1 to 5 µg of PS) are indicated in Table S1.

Table S1. Preparation of the calibration curve.

PS (µg)	Stock used (mg/L)	µL from the stock
0,1	20	5
0,2	20	10
0,5	200	2,5
1	200	5
2	200	10
5	2000	2,5

Validation

Validation of the method was performed in terms of instrumental repeatability, linearity, accuracy and precision, and limits of detection (LOD) and quantification (LOQ).

The instrumental repeatability was tested by injecting replicates of the PS standard at two masses, 0.1 and 2 µg. Relative standard deviation (RSD) was satisfactory (less than 10-15%).

Linearity was tested in the range 0.1 to 5 µg (higher masses saturate the detector), and adjusted to a quadratic regression with a regression coefficient greater than 0.99. Figure S1 shows the calibration curve obtained.

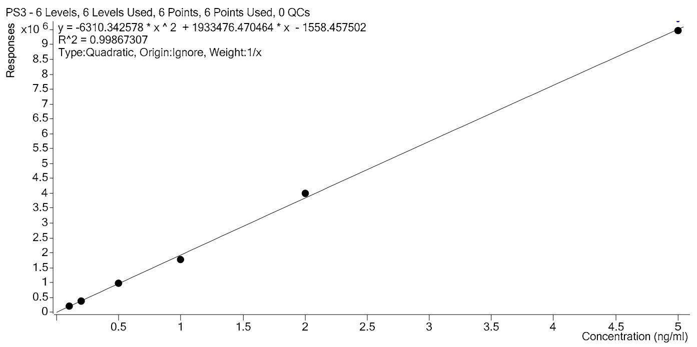

Figure S1. Calibration curve for PS from 0.1 to 5 µg.

The instrumental LOQ for PS was established at the lowest level of the calibration curve, i.e., the minimum value with an RSD less than 20%, a signal at least 10 times higher than the blank, and a linear trend with the other points. The LOD was established at a concentration in which the signal is at least three times higher than the signal of the blank.

Accuracy and precision were evaluated by studying recoveries from spiked samples subjected to the pre-treatment.

For permeate, validation was carried out with 100, 200, and 500 mL fortified with different concentrations of PS, from 0.2 to 1 mg/L (according to the filtered volume). An

average recovery of 124% was obtained for this process, and 8% of RSD from the three replicates.

For the activated sludge matrix, a volume of 100 mL was fortified at 10 mg/L and the average %R obtained was over 90%. Precision was not as good as RSD was 30%.

The limits of detection and quantification for each matrix were established according to the instrumental values, the volume of the sample treated and the possible presence of PS signal in the blank samples. Table S2 shows the LOQ and LOD established in each case.

Table S2. Instrumental LOQ and LOD established for each matrix.

	Volume (mL)	*LOQ (ug/ mL)*	*LOD (ug/ mL)*
Activated sludge	100	10	3
Permeate	100 – 500	0.04 – 0.2	0.013 – 0.067
